# Erosion and Corrosion Resistance Performance of Laser Metal Deposited High-Entropy Alloy Coatings at Hellisheidi Geothermal Site

**DOI:** 10.3390/ma14113071

**Published:** 2021-06-04

**Authors:** Andri Isak Thorhallsson, Francesco Fanicchia, Emily Davison, Shiladitya Paul, Svava Davidsdottir, Dagur Ingi Olafsson

**Affiliations:** 1 Innovation Centre Iceland, Árleynir 2-8, 112 Reykjavik, Iceland; svavada@nmi.is (S.D.); dagur@nmi.is (D.I.O.); 2 TWI Ltd., Granta Park, Cambridge CB21 6AL, UK; francesco.fanicchia@twi.co.uk (F.F.); emily.davison@twi.co.uk (E.D.); Shiladitya.Paul@twi.co.uk (S.P.)

**Keywords:** geothermal, high-entropy alloys, corrosion, erosion, H_2_S, CO_2_, O_2_, SEM, EDX

## Abstract

Geothermal process equipment and accessories are usually manufactured from low-alloy steels which offer affordability but increase the susceptibility of the materials to corrosion. Applying erosion-corrosion-resistant coatings to these components could represent an economical solution to the problem. In this work, testing of two newly developed laser metal deposited high-entropy alloy (LMD-HEA) coatings—CoCrFeNiMo_0.85_ and Al_0.5_CoCrFeNi, applied to carbon and stainless steels—was carried out at the Hellisheidi geothermal power plant. Tests in three different geothermal environments were performed at the Hellisheidi site: wellhead test at 194 °C and 14 bar, erosion test at 198 °C and 15 bar, and aerated test at 90 °C and 1 bar. Post-test microstructural characterization was performed via Scanning Eletron Microscope (SEM), Back-Scattered Electrons analysis (BSE), Energy Dispersive X-ray Spectroscopy (EDS), optical microscopy, and optical profilometry while erosion assessment was carried out using an image and chemical analysis. Both the CoCrFeNiMo_0.85_ and Al_0.5_CoCrFeNi coatings showed manufacturing defects (cracks) and were prone to corrosion damage. Results show that damage in the CoCrFeNiMo_0.85_-coated carbon steel can be induced by manufacturing defects in the coating. This was further confirmed by the excellent corrosion resistance performance of the CoCrFeNiMo_0.85_ coating deposited onto stainless steel, where no manufacturing cracks were observed.

## 1. Introduction

In geothermal power production, hot geothermal fluid from the earth’s crust is discharged from several or dozen wells where the geothermal energy from the fluid is utilized in steam turbines to produce mechanical and eventually electrical energy as a final energy product. During the processing of geothermal fluid from the initial to the final processing steps, the fluid flows through various equipment, including casing, piping, bends, valve housings, separators, heat exchangers, turbine blades, etc. Due to some corrosive chemical species and the high-velocity flow of the geothermal fluid, the equipment and accessories materials can be subject to erosion and corrosion, accelerating the material degradation rate and reducing the lifetime of the equipment and its accessories.

The extent of corrosivity of a geothermal environment can vary between geothermal systems due to significant variations in their thermodynamic and chemical properties [1]. The erosive and corrosive impact on the material depends on the fluid temperature, chemical composition, velocity, phase state of the geothermal fluid, the extent of scaling on the material surface, susceptibility of the material, etc. [2]. Previous corrosion performance studies of materials have reported corrosion and erosion-corrosion behaviors in tests that were conducted in both in situ geothermal environments [3,4,5,6] and simulated geothermal environments [7,8]. In recent years, studies of various newly developed metallic and composite protective coatings have been tested in a geothermal environment where different protective coatings were used: polysiloxane ferroferric oxide coatings have been shown to improve the corrosion resistance of carbon steel in geothermal pipelines [9] and chemical vapor deposited (CVD) titanium carbonitride coating has shown degradation due to formation of titanium chlorides in acidic geothermal brine [10], but CVD iron boride coating in a geothermal fluid containing H_2_S, CO, CO_2_, and chloride has shown good corrosion resistance performance [11]. Short-term testing of polytetral tetrafluoroethylene/(hexafluoropropylene coatings has shown some protective ability, but an indication of surface oxidation damage at 200 °C with high concentration of CO_2_ and NaCl in the geothermal fluid, Zirconia, and titanium oxide composite deposited with chemical liquid phase method has been shown to enhance the corrosion resistance of austenitic stainless steel in geothermal water [12].

Among alloys that could be classified as new coating materials are high-entropy alloys (HEA), a promising candidate for application in high-temperature geothermal environments where other more conventional alloys can be subject to erosion or corrosion damage. High-entropy alloys can also be deposited or coated [13] on less corrosion-resistant substrates, which can enhance the erosion and corrosion resistance of the more susceptible process and accessory equipment in a geothermal energy facility. In a geothermal environment, this relatively novel class of alloys has received considerable attention thanks to the good corrosion performance of the CoCrFeNiMo alloy [13,14]. Starting from this alloy, alloying elements could be added/removed to significantly improve the thermo-mechanical and corrosion performance in a geothermal environment. As an example, the addition of Cu to the solid solution of CoCrFeNiMo matrix affects the corrosion resistance of the alloy significantly, resulting in the formation of copper sulfide corrosion products in a H_2_S-containing environment [14]. The Al_0.5_CoCrFeNi alloy has been reported by Lin and Tsa [15] in other studies to have poor corrosion performance in a Cl^−^ containing environment, due to the attack of the chloride ion on the Al-rich phase in the alloy. Few or no studies have been reported, however, where the CoCrFeNiMo and Al_0.5_Co CrFeNi alloys were tested in a harsh erosive-corrosive environment and where the high-entropy alloys were produced via the laser metal deposition (LMD) methodology.

One of the properties that are sought after in the production of HEAs is enhanced mechanical strength of the material, which, in an addition to good corrosion resistance properties, can make the HEAs an optimal selection for a material application in a harsh, erosive-corrosive environment [16,17,18]. In the light of HEA application via LMD technology for geothermal applications, substrates such as carbon steels and stainless steels could be employed to evaluate the protection of components such as piping, casing, steam turbine, etc. For instance, it is well known that carbon steel application within the geothermal field involves a thick material wall design required to account for a uniform thinning (due to corrosion) of the material. Due to the enhanced performance of HEAs, the application of LMD coated HEAs on carbon steel could therefore reduce the corrosion rate and hence reduce material wall thickness requirements. Furthermore, the application of HEA coating could also extend the lifetime of the equipment in the corrosive geothermal environment.

In this study, HEA coatings of CoCrFeNiMo_0.85_ (Mo-HEA) and Al_0.5_CoCrFeNi (Al-HEA) materials were prepared with LMD on carbon steel and stainless steel substrates. The coatings were tested in three different geothermal environments to evaluate their corrosion and erosion resistance performance in geothermal environments. Two of the tests were performed in pure geothermal fluid from two different wells, while in the third test, oxygen was introduced in the geothermal fluid. This latter condition can be representative, for instance, of the axial seal system suction path. The effect of manufacturing defects on the corrosion performance of the coatings was evaluated by post-exposure analysis.

## 2. Materials and Methods

### 2.1. Testing Materials

Gas-atomized CoCrFeNiMo_0.85_ powder was produced under an argon atmosphere (HERMIGA 75/5 VI EAC, Phoenix Scientific Industries Ltd., Brighton, UK), with an estimated cooling rate of 105–106 °C/s. A powder of final size distribution −48 + 15 µm was finally obtained by employing mechanical sieving. Mechanically alloyed Al_0.5_CoCrFeNi powder was produced from powders of pure elements Co, Cr, Fe, Ni, and Mo (Laboratorium^®^, Bucharest, Romania), processed with a planetary ball mill (Fritsch-Pulverisette 6^®^, Idar-Oberstein, Germany) for an effective time of 210 min. Elemental powders were placed in a stainless steel vial with stainless steel balls in a 10:1 ball-to-powder weight ratio for this composition. The wet milling process was undertaken, in 2% n-heptane, to increase the alloying ratio and decrease the tendency of the powders to adhere to the balls or vials. From the overall batch of powder produced, a −56 + 20 µm size distribution was extracted by using mechanical sieving.

The laser cladding process was conducted using a Trumpf Trudisk 8002 (Trumpf GmbH, Ditzingen, Germany) with 5.3 kW disc laser system equipped with a TruControl 1000 controller (Trumpf GmbH, Ditzingen, Germany) and Trumpf BEO D70 processing optics (Trumpf GmbH, Ditzingen, Germany) with motor collimation. A Reis RV60-40 robot was employed to control the laser system, and an Oerlikon-Metco 10-C powder feeder (Oerlikon Metco, Pfaffikon, Switzerland) was used to control the powders. All the depositions were performed under an argon atmosphere to avoid the formation of oxide phases.

Carbon steel S235JR, stainless steel AISI 316L (316L), and stainless steel AISI 304L (304L) materials were employed as substrates. Carbon steel is normally used as a structural material in geothermal piping and casing, 316L is used as a structural material for a valve stem and turbine blades, and 304L is used as a structural material for turbine diaphragms. The CoCrFeNiMo_0.85_ (Mo-HEA) coating was deposited on the S235JR substrate (sample Mo-HEA-S235JR) and the 316L stainless steel substrates (sample Mo-HEA-316L). However, the Al-HEA coating was only deposited on the 304L substrate (sample Al-HEA-304L). Substrates of 50 mm × 25 mm × 6 mm dimensions were employed for the wellhead and aerated tests, while a 108 mm × 3 mm disc geometry was employed for the erosion test. Substrates were prepared by mechanical grinding with 60 grit paper followed by acetone degreasing before coating deposition. Three sample types were prepared for all the test locations. Both coatings, Mo-HEA and Al-HEA, had 800–1000 µm thickness. No post-treatment (grinding or polishing) was performed after the deposition of the coatings on the substrates. The chemical composition of the HEA coatings and the combination of coating and substrate in the test samples can be viewed in Table 1.

### 2.2. Test Equipment

#### 2.2.1. Wellhead Test

The corrosion performance tests were conducted in three separate locations at different conditions: at the wellhead of well HE-23, in an aerated pressure vessel connected to a separator fluid from several wells, and in an erosion-corrosion test unit connected to well HE-56. The HEA coatings were coated on the substrate samples in all the tests. One of the corrosion testing locations, specified onward in the paper as the “wellhead” test, was conducted at wellhead HE-23, where coupons were mounted in a test pipe connected to the wellhead. The wellhead test aimed to investigate the corrosion performance of the HEA coatings in an actual (deaerated) geothermal environment. Pictures from the wellhead, aerated, and erosion test equipment can be viewed in another paper by Thorhallsson et al. [19].

#### 2.2.2. Aerated Test

The second testing location, specified onward as the “aerated” test, was arranged such that separator fluid from several wells was led to a custom-made pressure vessel that also included drafted atmospheric gas at ambient pressure to simulate corrosive conditions that can occur in a geothermal turbine where oxygen ingression in the turbine assembly can be expected. In this testing equipment, geothermal fluid from the separator unit was mixed to ambient atmospheric gas, and due to the mixing, the test pressure was reduced down to 1 barA and temperature was reduced down to 90 °C. The same type of coupon sample was accommodated in the aerated test pressure vessel as was applied in the wellhead test.

#### 2.2.3. Erosion Test

The last test location, referred to hereafter as the “erosion” test, involved the application of HEAs-coated test plates that were accommodated in a custom-made test assembly that directs the flow of the geothermal fluid at high-velocity to a concentrated impact point (5 mm diameter), which hit the HEA coated test plates with expected erosive effect. The erosion test equipment was designed with a nozzle to spray the geothermal steam discharged from the wellhead of well HE-56 at high velocity onto a circular test plate sample at 90° angle in six test assemblies, each accommodating one test plate. As the geothermal fluid passed through the nozzle, the pressure decreased and the fluid was flashed, increasing its velocity. The velocity acceleration of the flashed fluid had a constant erosive-corrosive effect on the sample as droplets and particles hit the test sample surface. The equipment was designed in such a manner that the test plates were approximately 108 mm in diameter and 3 mm thick to fit well into the test units.

### 2.3. Test Conditions

For the chemical analysis of the geothermal fluid in wells HE-23 and HE-56, sampling and analysis of the liquid and condensable gas (gas that was condensed and analyzed in liquid state) and gaseous species in the fluid had to be conducted separately. Sampling and analysis of the liquid were only conducted from the aerated pressure vessel. The test conditions and chemical analysis (of the most corrosive species) of the fluids from the three locations can be viewed in Table 2 and the testing periods in Table 3, respectively.

### 2.4. Post Exposure Analysis

#### 2.4.1. Microstructural and Chemical Analysis

Microstructural analysis was done in an optical microscope Zeiss Axio (Zeiss, Oberkochen, Germany), a Supra 25, Scanning Electron Microscope (Zeiss, Oberkochen, Germany) equipped with Energy Dispersive X-ray Spectroscopy, with a Si (Li) X-ray detector, and Back-Scattered Electron (BSE) detector (Oxford Instruments, Abingdon, UK), and data were processed with AZtec software (Oxford Instruments, AZtec software version 3.3). The analysis was performed on the surfaces and in cross-section. The samples were prepared for analysis by cross-sectioning with a diamond wafering blade and then mounted in thermosetting phenol-formaldehyde resin (bakelite) and cast under pressure. The cross-sectioned samples were ground to 1000 grit with SiC abrasive paper and polished further with 3 µm and 1 µm diamond paste slurry and eventually polished with colloidal silica with 0.02–0.06 µm particle size. The microstructural analysis was applied to study the corrosion damage but also to assess the erosion damage on the surface. The pre-exposure analysis was done on the HEA coatings by the Welding InstitutE (TWI, Cambridge, UK) with SEM/BSE and EDX.

#### 2.4.2. Microscopic Erosion Assessment

The geometry of the erosion pits was evaluated by analyzing the cross-section of the coated test plates compared to the unexposed samples. Detached HEA coating material residuals on the surface or inside the scaling material were also used as an indicator for erosion-corrosion of the coatings.

#### 2.4.3. Macroscopic Erosion Assessment

**Figure 1:**
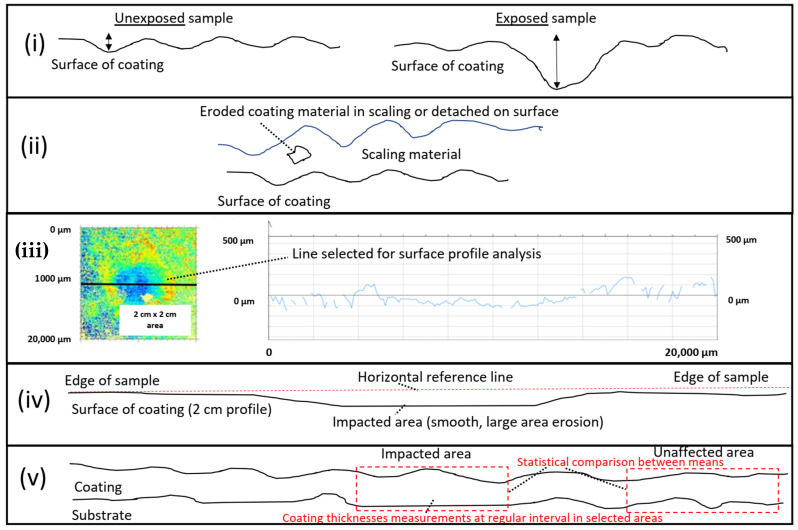
Methods for erosion assessment in our study: (**i**) erosion pit and shape comparison with an unexposed sample, (**ii**) coating material detached from the surface or within scaling, (**iii**) roughness measurements with optical profilometry, (**iv**) analysis with horizontal line adjacent to 20 mm length surface by image splining, and (**v**) statistical analysis of coating thicknesses in impacted area vs. unaffected area.

The extent of macroscopic erosion (erosion extending over a few mm) was evaluated, on the surface, by optical profilometry after exposure in the erosion test and with splining of SEM images. This analysis was conducted by applying a horizontal line adjacent to the flat surface outside of the impacted zone and to detect if any deviation was observed from the horizontal line in the impacted area as an erosion indicator. Finally, a statistical null hypothesis with a two-sided significance level of α = 0.025 of coating thicknesses measurements with a regular interval in the impacted area vs. coating thicknesses measurements with a regular interval in the unaffected area was attempted as another tool or erosion assessment. However, due to the extent of variability of the HEA coating thickness vs. the extent of erosion, this methodology was not conclusive. The methods for microscopic and macroscopic erosion assessments are summarized in Figure 1.

## 3. Results and Discussion

### 3.1. Wellhead Test

**Figure 2:**
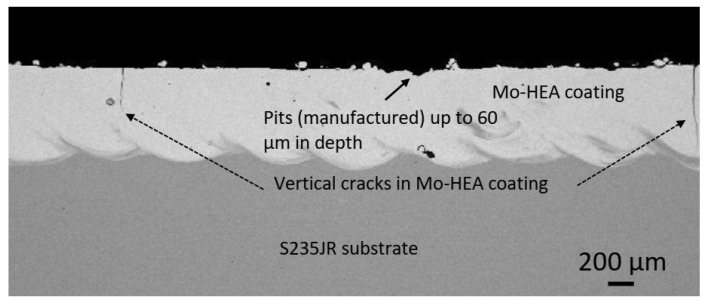
Unexposed sample Mo-HEA-S235JR.Vertical cracks were observed in the Mo-HEA coating on substrate S235JR, and variance on the coating surface, in the form of pits, was also observed.

The analysis of the unexposed samples revealed that significant variance was observed in the HEA coating thicknesses, between samples, and within each sample. This variance was mainly due to the coating-substrate profile variance. Manufacturing pits up to 50–60 µm in depth were also observed in the Mo-HEA coatings. The surface profile analysis showed that manufacturing pits were observed on all the unexposed samples, and manufacturing cracks were also observed in the coating of the Mo-MO-HEA-S235JR sample, as can be seen in Figure 2, but no cracks were though observed in the coating in sample Mo-HEA-316L.

**Figure 3:**
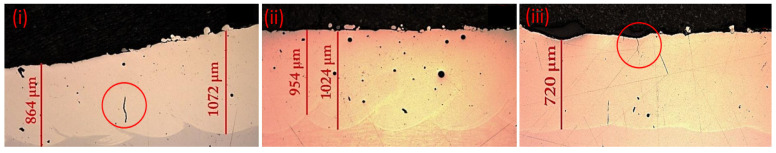
(**i**) Mo-HEA-S235JR with manufacturing crack in the coating, (**ii**) Mo-HEA-316L with no crack in the coating, and (**iii**) Al-HEA-304L samples with crack after the exposure.

In our analysis and for the evaluation for the extent of the erosive and corrosive effect of the geothermal environment on the samples, a comparison study between unexposed and exposed samples was an important analytical method due to the significant degree of variability on the surface of the as-received samples and the tested samples (samples not ground down or polished before testing). Cracks in coatings of samples Mo-HEA-S235JR and Al-HEA-304L were also observed, as seen in Figure 3. The cracks in the Mo-HEA and Al-HEA coatings were concluded to have formed in the manufacturing or during the deposition of the coatings on the substrates. The cracks in the Mo-HEA coating were likely formed due to high thermal input or thermal stress property difference between the Mo-HEA coating and the carbon steel substrate, and the cracking in the Al-HEA could be the result of internal stresses and hardness of the coating, but these assumptions are a subject for more thorough study and are not in the scope of this paper.

**Figure 4:**
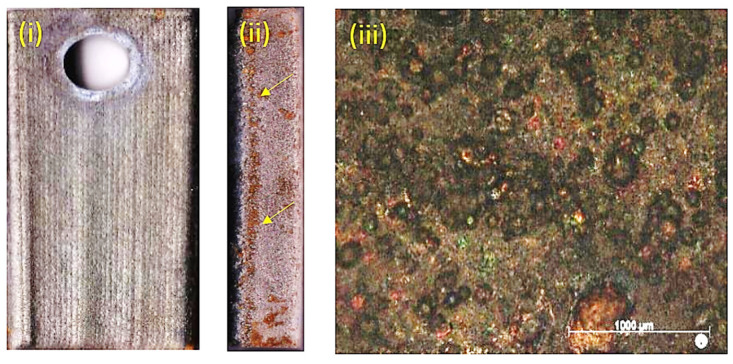
Mo-HEA-S235JR sample after the exposure in the wellhead test: (**i**) front face with negligible erosion damage observed, (**ii**) cross-section of the sample showing curvature and iron-based corrosion products in the coating-substrate interface, and (**iii**) magnified surface of the front face showing sphere-like unfused Mo-HEA powder on the surface.

In the testing at the wellhead of HE-23, the Mo-HEA coatings on the carbon steel and stainless steel substrates were not prone to corrosion damage apart from the manufacturing crack damage that was observed on the unexposed samples. A negligible corrosion effect was observed in the Mo-HEA coated samples in the surface analysis, but some curvature was observed in the Mo-HEA-S235JR sample. The curved shape of the sample could be due to the creeping of the sample that resulted from thermal stresses arising from the heating and cooling of the Mo-HEA-S235JR sample during the laser metal deposition of the Mo-HEA powder on the substrate. After the wellhead test, more brownish and reddish iron corrosion products were observed at the interface of the Mo-HEA coating and the carbon steel S235JR substrate in comparison with other locations on the substrate. The iron-based corrosion products formation could have formed during the test but may have also have formed after the test when wet samples were removed from the test chamber and exposed to the ambient atmosphere for a short period before drying and storage of the sample. The increased iron-based corrosion product formation on the Mo-HEA coating and the carbon steel, S235JR, substrate may also have been induced by the galvanic effect, i.e., the increased oxidizing effect of the noble Mo-HEA coating on the carbon steel substrate when the sample was wet, removed from the test chamber, and exposed to the ambient atmosphere. The iron-based corrosion products formed at the Mo-HEA coating and S235JR substrate in the curved sample can be viewed in Figure 4.

**Figure 5:**
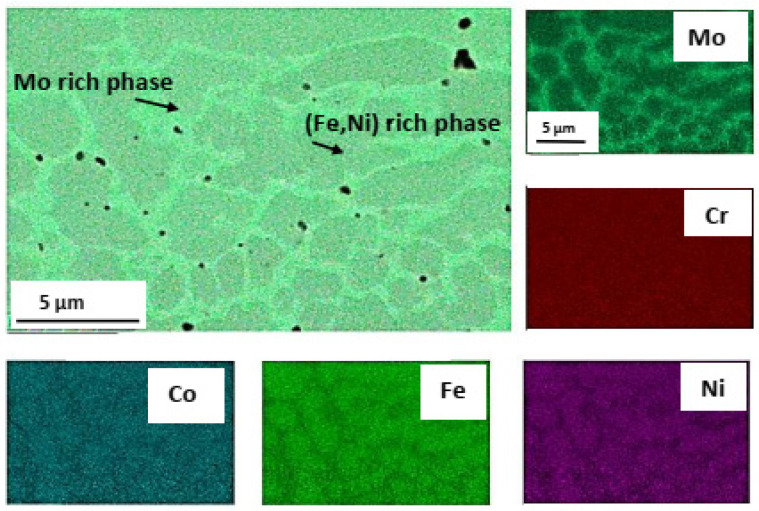
Pores (entrapped gas bubbles) were preferably observed in the Mo-rich phase in the Mo-HEA coating.

The galvanic sample was not observed in the other two samples, Mo-HEA-316L and Al-HEA-304L. In the Mo-HEA coating, two phases were apparent in the material, a Mo-rich phase and (Fe,Ni)-rich phase. Some porosity was preferably observed in the Mo-rich phase, as seen in Figure 5, indicating a higher melting point of the Mo-rich phase in comparison with the (Fe,Ni)-rich phase.

**Figure 6:**
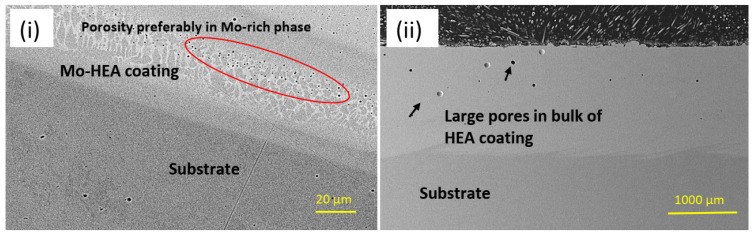
(**i**) A high number of pores was observed in the Mo-HEA coating at the substrate but (**ii**) at a lower frequency and larger volumes in the bulk coating and closer to the surface.

Porosity appears to be localized in the bulk of the coating, but smaller pores were observed in the area closer to the substrate, as seen in Figure 6.

Despite the manufacturing defects in the Mo-HEA coating, i.e., cracks and entrapped gas bubbles, no adhesion damage was observed on either the carbon steel and stainless steel substrates, both in the as-deposited state and after exposure.

**Figure 7:**
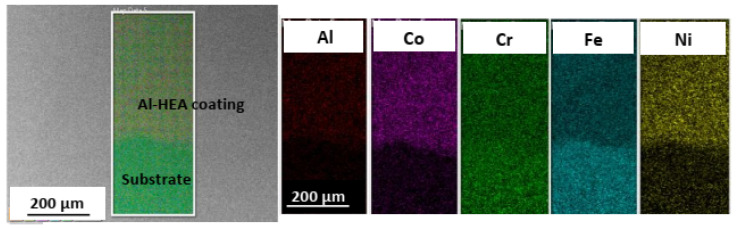
Elemental mapping of the boundary between Al-HEA coating and the 304L substrate.

The Al-HEA coating in sample Al-HEA-304L contains Al but not Mo as in the Mo-Coating. In the comparison of Al-HEA coating with the Mo-HEA coating, a more homogeneous microstructure can be observed in the Al-HEA coating material, with the appearance of a more single-phase matrix as seen in Figure 7.

**Figure 8:**
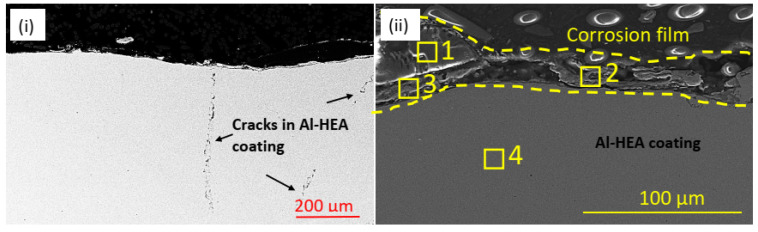
(**i**) Manufacturing cracks and (**ii**) oxide-rich corrosion products (and some sulfides) on the surface on the exposed in the Al-HEA coating in sample Al-HEA-304L in the wellhead test.

The Al-HEA coating was prone to corrosion damage in the wellhead test in addition to the manufacturing cracks in the coating. Oxide-rich corrosion film in the thickness range 20–40 µm was observed on the surface as seen in Figure 8 and Table 4, indicating that the oxidation could be due to a reaction with oxygen-containing molecules in the geothermal fluid. Some sulfide-based corrosion products were also detected in the corrosion film.

### 3.2. Aerated Testing

**Figure 9:**
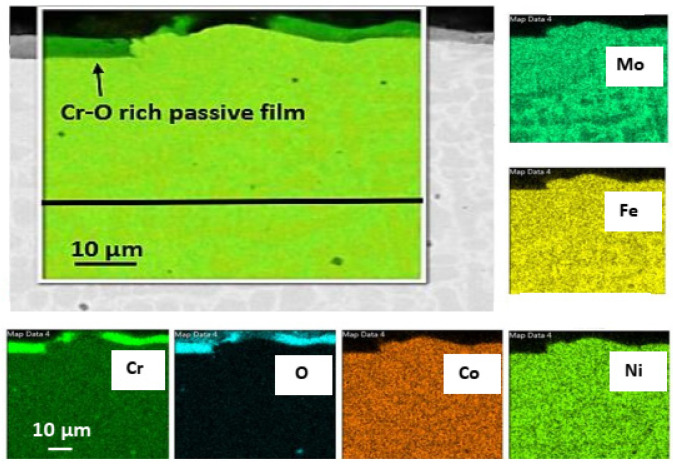
Elemental mapping of the Mo-HEA coating showing the extent of the discontinuous passive film formed on the coating.

For the Mo-HEA coating on the 235JR and 316L substrates, no corrosion damage was observed after the exposure in the aerated testing. It was, however, observed that the Mo-HEA coating had formed a partial, Cr-O rich, passive layer on the surface as seen in Figure 9.

The oxide passive film resulted from the exposure to the oxygen in the aerated fluid, but to some surprise, the Cr-O film was always discontinuous on the surface and did not cover the whole surface of the Mo-HEA coating. The reason for the discontinuous oxide layer on the surface is not fully understood, but one explanation is that Cr concentration gradients that are present in the bulk coating near the surface and sluggish diffusion rate of Cr from the high entropy material to the surface could result in slow oxide layer formation in some locations. Hence, a propagating growth of the oxide film to become more continuous on the surface could be attained by longer exposure periods in the aerated fluid, but this should be verified with longer exposure periods. Another explanation could be that the oxide film was at some point continuous, but the oxide film is brittle, and some parts of it flaked off and were removed by the fluid during the exposure.

**Figure 10:**
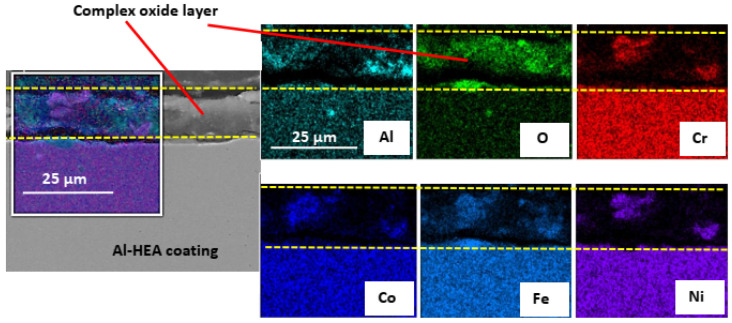
In some cases, the complex oxide coatings formed on the Al-HEA coating surface was continuous after the exposure to the aerated fluid.

The oxide layer formed on the Al-HEA coating had different composition and structure in comparison with the Mo-HEA oxide layer after the aerated test. The oxide layer on Al-HEA coating was found to have more corrosion damage characteristics rather than passive layer behavior, i.e., not with compact structure. The Al-HEA oxide layer was in some cases detached from the bulk material, and the layer was composed of oxides from all the elements in the HEA as seen in Figure 10.

**Figure 11:**
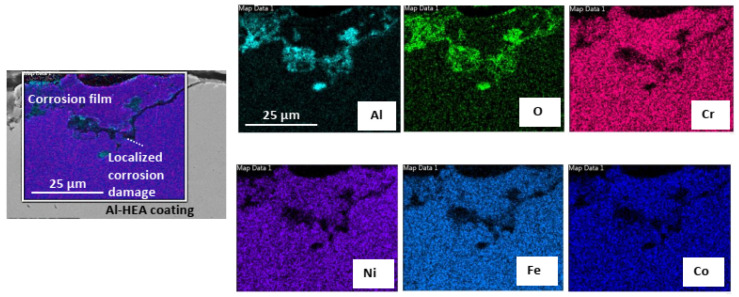
Elemental mapping of localized corrosion damage observed on the surface of exposed AL-HEA coating in the aerated test.

In addition, localized corrosion damage enriched in Al-O was also observed on the surface of the Al-HEA coating in locations where the complex oxide film had not formed to a significant extent as seen in Figure 11. Localized oxide corrosion forms were observed in the aerated test but not in the wellhead test.

**Figure 12:**
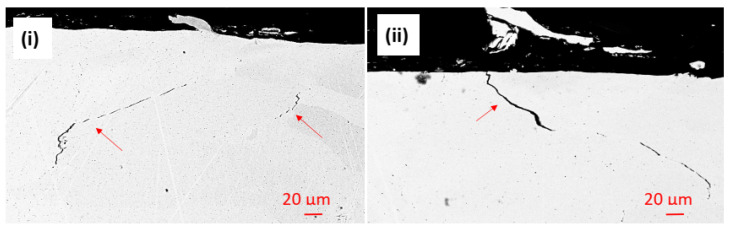
Cracks were observed in (**i**) the bulk and (**ii**) at the surface of the Al-HEA coating in the aerated testing.

The Al-O-rich localized damage might suggest that the oxidation of Al has faster kinetics than the oxidation of the other elements. This localized corrosion behavior also strengthens the assumption that the complex oxide film is not passive. Cracks were also observed in the Al-HEA coating in the sample Al-HEA-304L after the exposure in the aerated environment as seen in Figure 12.

It was noted that the shape and appearance of the cracks in the aerated testing were more curved and thinner in comparison with the cracks observed in the Al-HEA coating after the exposure in the wellhead test. The effect of a difference in temperature in the aerated and the wellhead, and hence the extent of thermal stresses, could explain to some extent this variance in cracking behavior. However, no corrosion products were observed in the cracks in the Al-HEA coating, and hence the cracking was concluded to have formed due to internal stresses in the coating.

### 3.3. Erosion Testing

**Figure 13:**
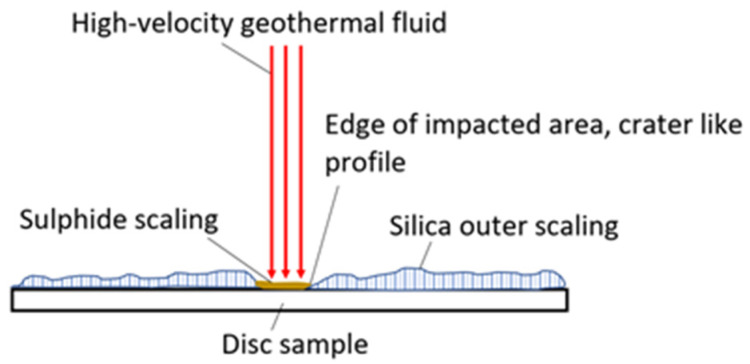
The most common scaling (inner sulfide and outer silica scaling) profile on samples after exposure in erosion testing.

Due to the large extent of scaling and material deposition on the samples after the exposure, the surface roughness measurements were only an indicator of the scaling/precipitation landscape rather than on the extent of erosion of the HEA coatings. As a result, the surface roughness measurements were inconclusive in our analysis. In the erosion test, the high-velocity fluid hit the HEA coatings deposited on a substrate test plate. Generally, the impacted area (where high-velocity fluid hit the surface) was covered with sulfide scaling, but outside of the impacted area, silica scaling was more profound, as exhibited in Figure 13.

**Figure 14:**
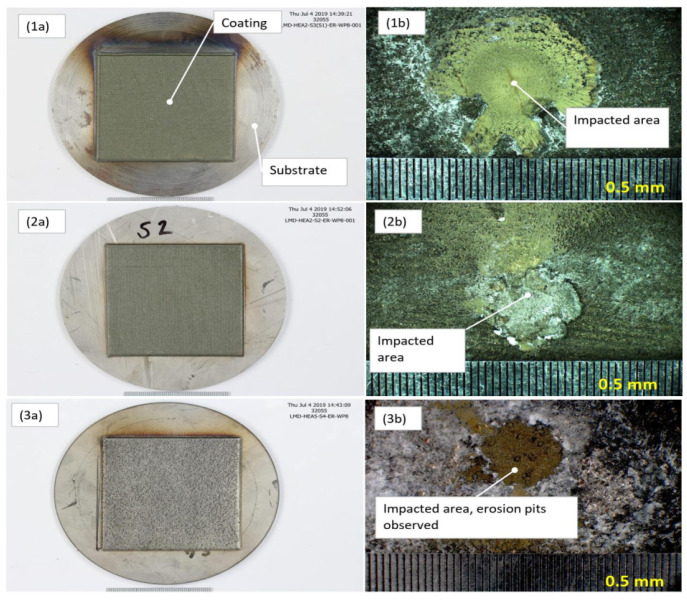
Erosion disc test samples. (**1a**) Mo-HEA-S235JR test plate before exposure and (**1b**) the impacted area after exposure. (**2a**) Mo-HEA-316L test plate before exposure and (**2b**) the impacted area after exposure. (**3a**) Al-HEA-304L test plate before exposure and (**3b**) the impacted area after exposure.

After the erosion test exposure, the samples were covered with sulfide and silica scaling. No evident erosion effect was observed on the samples at low magnification in the stereoscope/microscope, but erosion pits could though be observed in the Al-HEA coating, as seen in Figure 14.

Roughness analysis with optical profilometry was conducted on the samples after the exposure in the erosion test. In the surface profile analysis, it was observed that the surface profile was dominated by the scaling and deposits on the surface but not by shallow erosion pits. As a result, the erosion assessment with the surface profile analysis was inconclusive.

The variance in the HEA coating thicknesses was quite significant and exceeded the erosion effect observed for all the samples. Hence, the estimation of erosion with the null hypothesis method could not be conclusive for any of the analyses.

**Figure 15:**
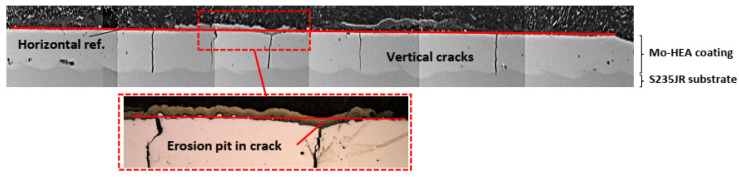
The surface profile of Mo-HEA coating in the Mo-HEA-S235JR sample after the exposure in the erosion testing. The erosion pit was concluded to have formed in one of the vertical cracks in the Mo-HEA coating.

The results of the corrosion performance of the Mo-HEA coatings in the erosion testing were in good agreement with the results from the wellhead and aerated testing; i.e., the Mo-HEA coating was not prone to electrochemical corrosion. In the erosion test, however, the Mo-HEA coating was prone to erosion damage in one vertical crack at the surface, as can be seen in Figure 15.

**Figure 16:**
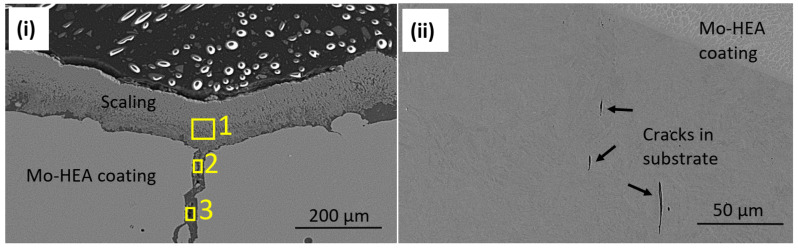
(**i**) Analysis of scaling/deposits in the eroded crater area and (**ii**) vertical microcracks were observed in the carbon steel substrate, at the coating interface, in the MO-HEA-S235JR sample.

Because the erosion pit (no pit observed in the unexposed sample) was observed in the center of a vertical crack in the Mo-HEA coating, it was concluded that the crack had induced some turbulence flow of the fluid when it hit the surface of the crack and hence increased the erosion effect of the fluid, resulting in the formation of a significant erosion pit in the crack. Detailed analysis of the erosion pit in the crack showed that sulfide and silica scaling, but no corrosion products, were in the crack. The sulfide content in our analysis was associated with high Fe and Cu content in the scaling. The smooth surface on erosion pits also implies that erosion occurred on the surface on the vertical crack as seen in Figure 16i and Table 5.

In the Mo-HEA-S235JR sample, some vertical microcracks (Figure 16ii) were observed in the S235JR substrate at the Mo-HEA coating and S235JR interface that likely formed during the LMD process i.e., manufacturing. The difference in thermal expansion properties and hence thermal stresses arising in the cooling after the LMD process could explain the vertical microcracks in the S235JR substrate to some extent.

**Figure 17:**
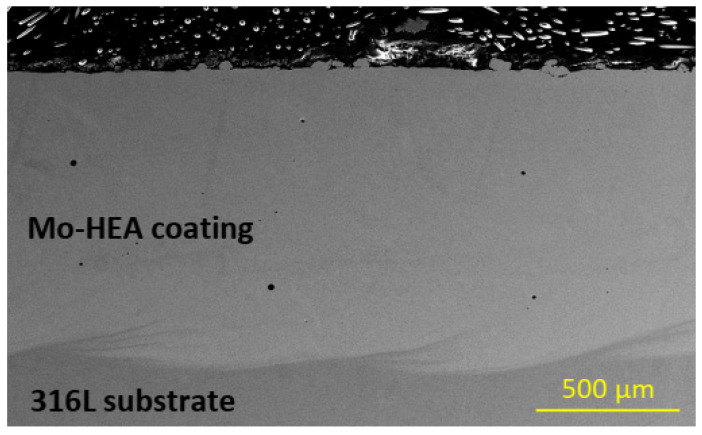
No cracking or erosion damage was observed in the Mo-HEA-316L sample in the erosion testing.

The Mo-HEA coating in the Mo-HEA-316L sample was not prone to any cracking in the erosion testing, as can be seen in Figure 17. This result implies that the stainless steel substrate is more compatible with the Mo-HEA coating in regard to the laser metal deposition method. The Mo-HEA coating was not susceptible to any corrosion damage in the test, which shows that the Mo-HEA coating has good erosion resistance when no cracking is inducing the erosion effect on the surface as concluded from the erosion observed in Mo-HEA coating on a carbon steel substrate.

**Figure 18:**
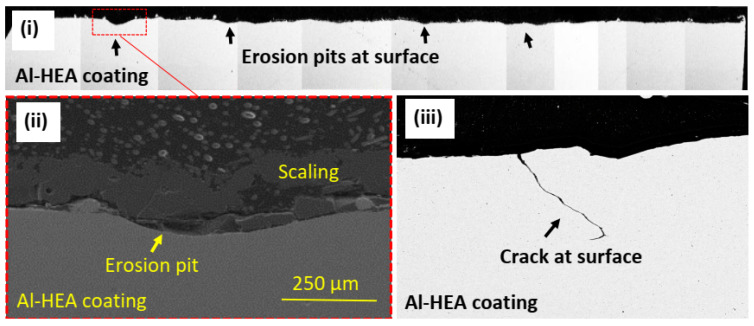
(**i**) Surface profile (BSE image) of exposed Al-HEA-304L sample with erosion pits at the surface, (**ii**) one erosion pit at high magnification (SEM image), and (**iii**) crack observed eroded in Al-HEA coating (BSE image) at the surface.

Cracking of the Al-HEA coating was, however, observed in the Al-HEA-304L sample in the erosion testing. The erosion-corrosion effect was evident in the impacted area, and the extent of erosion on the Al-HEA-304L sample could be observed on a large area in the surface profile, a couple of erosion pits with significant depth and smooth erosive shape were observed on the AL-HEA coating, as seen in Figure 18.

**Figure 19:**
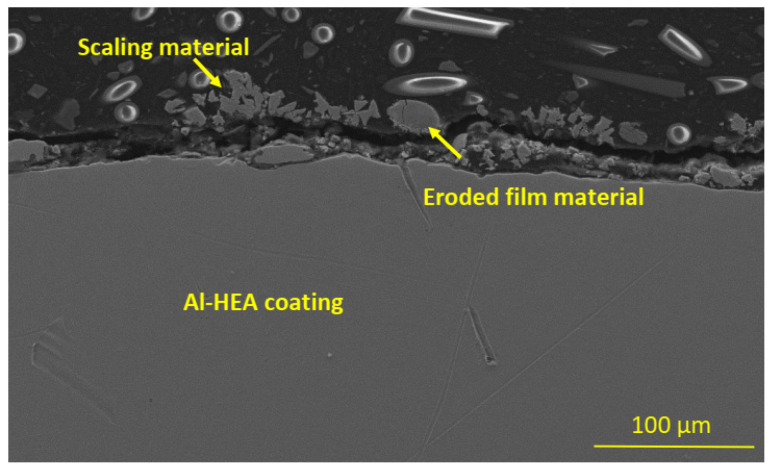
Eroded, detached AL-HEA material was observed at the surface in the sample AL-HEA-SS in the erosion test.

The erosion-corrosion damage in the Al-HEA coating was also observed by the detached, eroded material residuals on the surface, among the scaling and deposited material from the geothermal fluid, as can be seen in Figure 19. This observation further strengthens the conclusion that Al-HEA is prone to erosion in the testing environment.

## 4. Conclusions

In our study, two high-entropy coatings were tested in three different geothermal environments. High-entropy alloy (HEA) coatings of CoCrFeNiMo_0.85_ (Mo-HEA) were deposited using laser metal deposition (LMD) onto carbon steel (S235JR) and stainless steel (316L) substrates, and Al_0.5_CoCrFeNi (Al-HEA) coating was deposited using LMD onto stainless steel (304L) substrate. Erosion and corrosion resistance performance of the systems was evaluated after exposure in three different environments at the Helliseidi geothermal power plant: wellhead, aerated, and erosion test. In-depth metallographic examination and optical profilometry were performed to obtain information on the erosion damage and growth of the corrosive scale. The research has highlighted the following main findings:

- The LMD CoCrFeNiMo_0.85_ coating was prone to manufacturing cracking, in some cases throughout the coating, when deposited on carbon steel substrate, but not on stainless steel. Wide manufacturing cracks are concluded to enhance the fluid-induced erosion effect for the CoCrFeNiMo_0.85_ coating in the vicinity of the cracks.
- The LMD Al_0.5_CoCrFeNi coating was, in general, more susceptible to corrosion and erosion than the CoCrFeNiMo_0.85_ alloy in the geothermal environment tested. The Al_0.5_CoCrFeNi coating was prone to general and localized corrosion damage. In the wellhead test, oxygen and sulfide-rich corrosion products were observed, but an oxygen-rich corrosion layer was observed in the aerated test. In the erosion test, >100 µm-deep erosion pits were observed after the 90-day erosion test period.

From our results, it is concluded that LMD-manufactured CoCrFeNiMo_0.85_ coating could be a promising erosion- and corrosion-resistant coating candidate on less erosion-corrosion-resistant materials for application in geothermal environments. The formulation and production of the CoCrFeNiMo_0.85_ coating, however, need to be further optimized to prevent cracking of the CoCrFeNiMo_0.85_ coating on a carbon steel substrate. It is concluded that an Al_0.5_CoCrFeNi coating is not suitable for application as a coating material in a geothermal environment.

## Figures and Tables

**Table 1:** HEA coatings compositions, and coating–substrate combinations.

Sample Designation	Coating Composition [mol. Ratio]	Substrate
Mo-HEA-S235JR	CoCrFeNiMo_0.85_	Carbon steel (S235JR)
Mo-HEA-316L	CoCrFeNiMo_0.85_	Stainless steel (AISI 316L)
Al-HEA-304L	Al_0.5_CoCrFeNi	Stainless steel (AISI 304L)

**Table 2:** Test conditions at the three test locations.

Location	Wellhead Test	Aerated Test	Erosion Test	Unit
Temperature	194	90	198	°C
Pressure	13.6	1	14.9	barA
**Condensed Gas Analysis:**				
Cl^−^	62.1	-	161.3	mg/kg
SO_4_	58.4	-	14.2	mg/kg
SO_2_	488	-	672.4	mg/kg
Sr	0.0356	-	-	mg/kg
Ti	<0.002	-	-	mg/kg
**Gas Analysis:**				
CO_2_	15,017	-	2178	mg/kg
H_2_S	1363.4	-	562	mg/kg
CH_4_	-	-	4	%Vol.
H_2_	-	-	82.4	%Vol.
Cl^−^	62.1	-	161	mg/kg
F	0.337	-	1.2	mg/kg
SO_4_	58.44	-	10	mg/kg
**Liquid Analysis:**				
CO_2_	123	-	36	mg/kg
Cl	-	0.35	-	mg/kg
F	0.337	0.072	-	mg/kg
H_2_S	33.7	-	68.4	mg/kg
SO_4_	-	3.7	-	mg/kg
Conductivity	550	-	1030	µS/cm
pH (at RT)	8.26	4.9	9.1	

**Table 3:** Test locations and the relevant testing periods.

Test Batch	Test Location/Setup	Testing Period [Days]
Wellhead	Wellhead HE-23	90
Aerated	Separator fluid + air	90
Erosion	Wellhead HE-56	60

**Table 4:** Elemental EDX analysis from locations in Figure 8ii.

Location	Element (wt.%)										
	O	Na	Al	Si	S	Cr	Ca	Cr	Fe	Co	Ni
1	46.0	-	52.7	-	-	-	0.2	1.1	-	-	-
2	2.6	-	4.3	-	2.1	-	-	12.5	33.3	18.6	26.7
3	9.8	1.3	4.6	0.3	5.7	-	-	14.7	26.8	16.2	20.7
4	-	-	3.1	-	-	-	-	15.2	37.6	19.1	24.9

**Table 5:** Elemental XEDS analysis from locations in Figure 16i.

Location	Element (wt%)										
	O	Na	Al	Si	S	K	Ca	Cr	Fe	Cu	Ag
1	19.3	0.9	2.3	10.6	22.2	0.8	0.5		21.3	21.2	1.1
2	11.5	-	1.3	5.8	27.7	0.5	0.3	0.3	28.3	22.4	2.1
3	51.3	2.6	7.6	33.5	-	2.9	1.7	0.2	0.3	-	-

## Data Availability

Data available on request due to restrictions eg privacy or ethical, The data presented in this study are available on request from the corresponding author.
